# Supplementary material for: Association between oral microbiome and seven types of cancers in East Asian population: a two-sample Mendelian randomization analysis
Source: Front Mol Biosci. 2023 Nov 21;10:1327893. doi: 10.3389/fmolb.2023.1327893 (PMC10702768; doi:10.3389/fmolb.2023.1327893)
Supplement: Supplementary file 1 [file DataSheet1.ZIP › Supplementary Materials/MR plots of saliva/saliva═╝/Prostate cancer/pheno.2859_to_prostate cancer_leave_one_out.pdf]

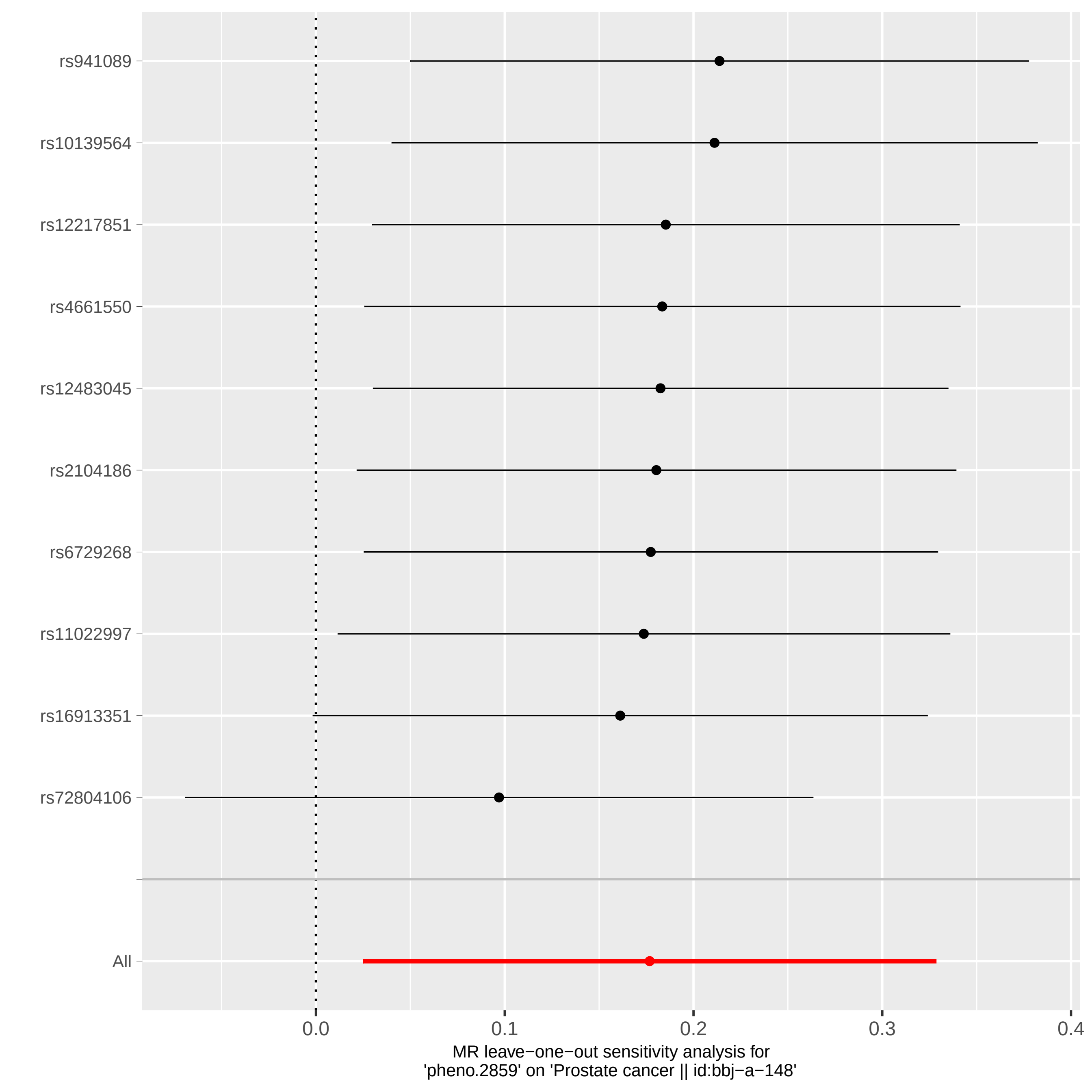
rs941089
rs10139564
rs12217851
rs4661550
rs12483045
rs2104186
rs6729268
rs11022997
rs16913351
rs72804106
All
0.0
0.1
0.2
0.3
0.4
MR leave−one−out sensitivity analysis for
'pheno.2859' on 'Prostate cancer || id:bbj−a−148'
